# Diaphragmatic rupture with right colon and small intestine herniation after blunt trauma: a case report

**DOI:** 10.1186/1752-1947-4-289

**Published:** 2010-08-24

**Authors:** Mirko Muroni, Giuseppe Provenza, Stefano Conte, Andrea Sagnotta, Niccolò Petrucciani, Ivan Gentili, Tatiana Di Cesare, Andrea Kazemi, Luigi Masoni, Vincenzo Ziparo

**Affiliations:** 1 Department of General Surgery, La Sapienza University of Rome, Second School of Medicine, St. Andrea Hospital, via di Grottarossa 1035, 00189 Rome, Italy

## Abstract

**Introduction:**

Traumatic diaphragmatic hernias are an unusual presentation of trauma, and are observed in about 10% of diaphragmatic injuries. The diagnosis is often missed because of non-specific clinical signs, and the absence of additional intra-abdominal and thoracic injuries.

**Case presentation:**

We report a case of a 59-year-old Italian man hospitalized for abdominal pain and vomiting. His medical history included a blunt trauma seven years previously. A chest X-ray showed right diaphragm elevation, and computed tomography revealed that the greater omentum, a portion of the colon and the small intestine had been transposed in the hemithorax through a diaphragm rupture. The patient underwent laparotomy, at which time the colon and small intestine were reduced back into the abdomen and the diaphragm was repaired.

**Conclusions:**

This was a unusual case of traumatic right-sided diaphragmatic hernia. Diaphragmatic ruptures may be revealed many years after the initial trauma. The suspicion of diaphragmatic rupture in a patient with multiple traumas contributes to early diagnosis. Surgical repair remains the only curative treatment for diaphragmatic hernias. Prosthetic patches may be a good solution when the diaphragmatic defect is severe and too large for primary closure, whereas primary repair remains the gold standard for the closure of small to moderate sized diaphragmatic defects.

## Introduction

Traumatic rupture of the diaphragm is an uncommon condition. It occurs in 0.8 to 5% of patients admitted to hospital with thoracoabdominal trauma. The etiologic factors are blunt trauma (for example, in motor vehicle accidents) and penetrating trauma [1]. The organs most commonly involved in right-sided diaphragmatic hernias are the colon, omentum, small intestines and liver.

Chest radiography and computerized tomography is the most effective method for diagnosis of traumatic diaphragmatic rupture [2]. Treatment is surgical, with reduction of the viscera and simple repair of the diaphragm with non-absorbable suture.

## Case presentation

A 59-year-old Italian man presented with abdominal pain localized in the right upper quadrant, constipation and vomiting for longer than one week. The patient had inconstant symptoms including shortness of breath and dyspnea. His medical history included right-sided rib fractures in a motor vehicle crash seven years previously.

**Figure 1:**
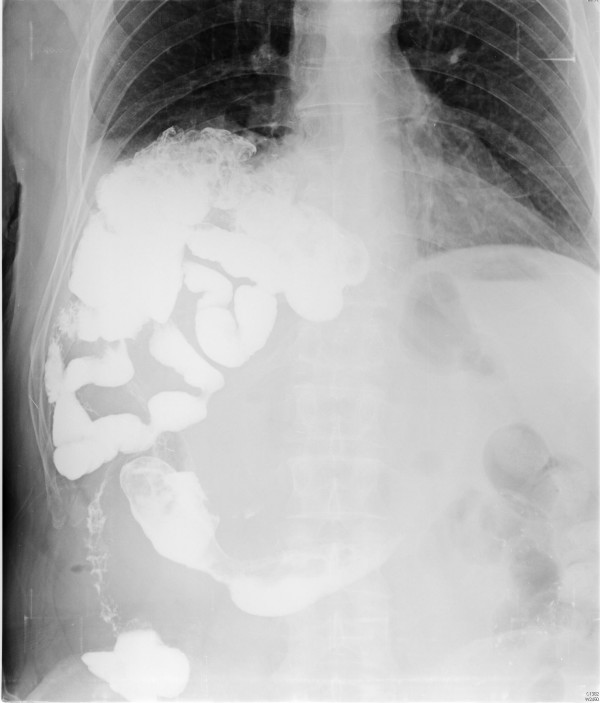
**Barium study showing small bowel and right colon herniation into the right hemithorax behind the liver**.

**Figure 2:**
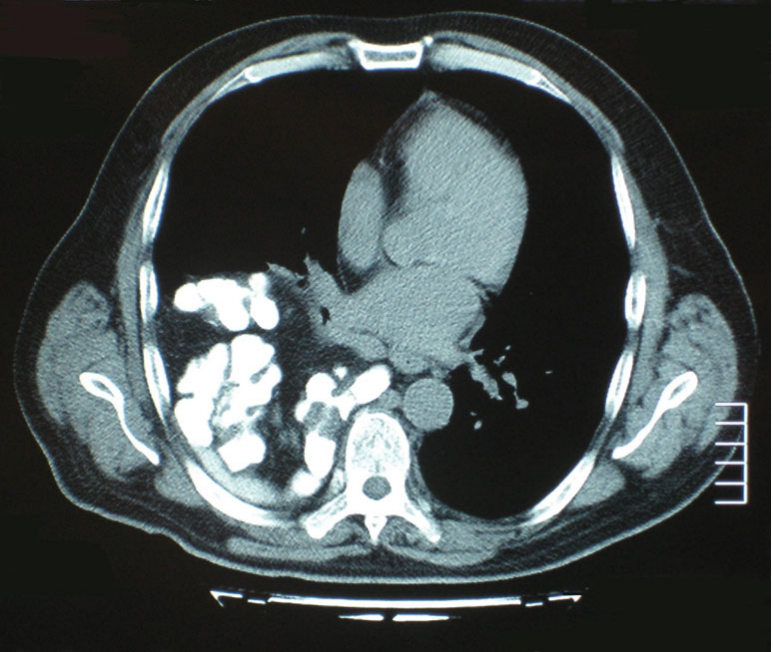
**Computed tomography image showing the right diaphragmatic hernia**.

On physical examination, bowel sounds were present in the right hemithorax on auscultation. A chest X-ray showed elevation of the right hemidiaphragm elevation, with a portion of the colon and the small intestine transposed in the right hemithorax as a diaphragmatic rupture. A barium study showed small bowel and right colon herniation into the right hemithorax, passing behind the liver (Figure 1). Computed tomography (CT) scan confirmed the diaphragmatic herniation (Figure 2).

**Figure 3:**
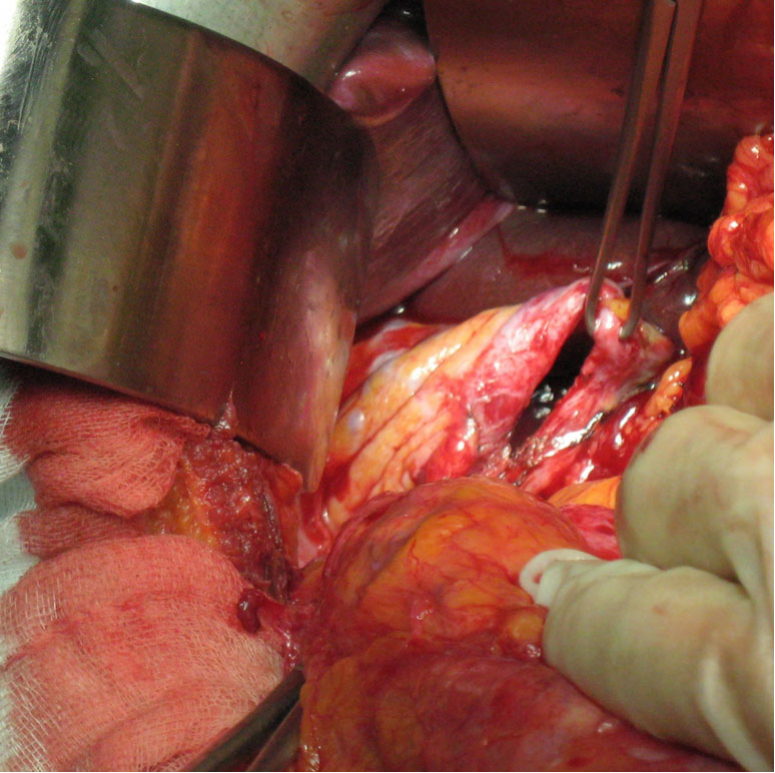
**An intraoperative photo showing the diaphragm defect**.

The patient underwent laparotomy, and herniation of the right colon and small intestine (40 mm in length) was seen. There were no ischemic changes or perforation, but the colon was slightly edematous. No resection of any part of the intestinal tract was necessary. The colon and the small intestine were reduced into the abdomen. As usual in traumatic lesions, there was absence of the hernial sack: the hernia opening was only 50 mm in length, (Figure 3). The hernia opening was repaired with interrupted non-absorbable sutures; placement of a polymeric prosthetic mesh was not required at the time of the intervention. A drain was placed in the right side of the thorax. The operating time was 45 minutes. The thoracic drain was removed on the third post-operative day and the patient was discharged on the fifth postoperative day.

## Discussion

Blunt diaphragmatic rupture is a rare event. Because of its low incidence and presence of associated injuries, early diagnosis is difficult [3]. Usually the diaphragmatic injury can be traced back to a violent force applied to the abdomen or chest.

We diagnosed our patient with diaphragmatic rupture seven years after the blunt trauma (motor vehicle crash) and right rib fractures had occurred. The patient's symptoms included abdominal pain and vomiting in the weeks leading up to presentation. Routine hematologic and biochemical investigations were normal. The diaphragmatic hernia was diagnosed by chest X-ray, which showed diaphragm elevation, with a gas shadow in the lower chest that was due to a portion of the colon and the small intestine being transposed into the right hemithorax. The CT scan confirmed the diagnosis. During the procedure, the defect in the posterolateral area of the diaphragm was found to be quite small, and the herniated right colon and small intestine were reduced into the abdomen without complications.

This was an unusual case of traumatic right-sided diaphragmatic hernia. Because the right diaphragm is protected by the liver, such injuries almost exclusively involve the left diaphragm [4-6]. Diaphragmatic ruptures may be revealed many years after the initial trauma, presenting as abdominal visceral herniation and complications such as strangulation and perforation [7].

Missed blunt diaphragmatic rupture results in herniation of the abdominal organs into the chest due to the abdominothoracic pressure gradient, which progressively enlarges the diaphragm defect. Progressive herniation results in chronic abdominal or/and chest pain, constipation, strangulation and perforation of the abdominal viscera, with shortness of breath, dyspnea, and respiratory infections due to compression of the lung on the affected side. Although very rare, a colopleural fistula through a diaphragmatic hernia has been described in the literature [8].

There are various surgical approaches for diaphragmatic hernias repair. Mesh patches are widely used. Polytetrafluoroethylene (Gore-Tex), polyethylene terephthalate (Dacron) and polypropylene are the most common materials used in prosthetic patches to repair large diaphragmatic defects that are not amenable to primary repair. Recently, some authors suggested the use of a newer biologic material which is composed by a sheet of collagen derived from porcine dermis [9]. However, there are cases in the literature reporting patch infection and hernia recurrence after the use of a mesh [10,11], thus we believe that primary repair with non-absorbable sutures is the best alternative for diaphragm repair, as it reduces infection risk and the costs of the procedure.

## Conclusions

Suspicion of diaphragmatic rupture in a patient with multiple trauma injuries contributes to an earlier correct diagnosis. Early diagnosis is very important for appropriate surgical management, reducing the risks of visceral strangulation and its complications. Surgical repair remains the only curative treatment for diaphragmatic hernias because such hernias are invariably associated with strangulation. Primary repair with non-absorbable sutures remains the gold standard for the closure of small to moderate sized defects. Patients with large defects may require patch closure with a mesh, but these can carry risks.

## Competing interests

The authors declare that they have no competing interests.

## Consent

Written informed consent was obtained from the patient for publication of this case report and any accompanying images. A copy of the written consent is available for review by the Editor-in-Chief of this journal.

## Authors' contributions

MM conceived the idea, wrote the manuscript, and performed the literature search. GP, SC, IG, TDC, NP assisted the literature search. AS and AK took the intraoperative photos. LM and VZ reviewed the manuscript. All authors have read and approved the final manuscript.
